# Increasing the Awareness of Animal Welfare Science in Marine Mammal Conservation: Addressing Language, Translation and Reception Issues

**DOI:** 10.3390/ani11061596

**Published:** 2021-05-28

**Authors:** Isabella L. K. Clegg, Rebecca M. Boys, Karen A. Stockin

**Affiliations:** 1 Animal Welfare Expertise, 18 Melrose Parade, Sydney 2031, Australia; 2 Cetacean Ecology Research Group, School of Natural and Computational Sciences, Massey University, Auckland 0745, New Zealand; r.boys@massey.ac.nz; 3 Animal Welfare Science and Bioethics Centre, Massey University, Private Bag 11 222, Palmerston North 4442, New Zealand

**Keywords:** animal welfare science, conservation biology, marine mammals, wild animal welfare

## Abstract

**Simple Summary:**

Traditional conservation strategy focuses on population-level effects. However, the rapidly evolving scientific discipline of animal welfare science, in conjunction with growing societal expectations around the value of individuals, is prompting change in conservation. Despite growing recognition of this approach in terrestrial conservation efforts, limited application of animal welfare science to marine mammals has been observed. To investigate the factors underlying this disparity, we undertook an initial “Welfare in the Wild” workshop at the 32nd European Cetacean Society conference (La Spezia, Italy) to explore expert opinion on this topic. Secondly, we analysed the English language peer-reviewed literature to assess to what extent marine mammal welfare research is reported. The results of the workshop reveal a range of views about the scientific discipline of animal welfare science, with participants’ definitions varying depending on their disciplinary expertise. Meanwhile, the extensive literature review spanning 1950 to July 2020 revealed extremely low reporting of research related to welfare in the context of marine mammals, with only 0.96% (*n* = 299) of all published articles on marine mammal taxa (*n* = 31,221) featuring the word *welfare* in either the title, abstract or keywords. This highlighted a need to explore differences and distil common themes. Here we suggest practical solutions to the language, translation and reception issues of this cross-disciplinary collaboration between animal welfare science and marine mammal conservation.

**Abstract:**

Integrating welfare principles into conservation strategy is an emerging synthesis that encourages consideration of individual animals’ quality of life in research, policies and law. However, these principles have gained limited traction in marine compared to terrestrial animal conservation. This manuscript investigates several factors that may be contributing to this disparity. In order to gauge current understanding of animal welfare science principles by marine mammal researchers and other stakeholders, a “Welfare in the Wild” workshop was convened at the 32nd European Cetacean Society conference (La Spezia, Italy, April 2018). The workshop was attended by 30 participants who completed pre- and post-workshop surveys on animal welfare principles. The survey results highlight a range of different views about exactly what animal welfare science is and how it can be applied to marine mammals. Specifically, participants’ definitions appeared to vary depending on the type of employment or research they engaged in, indicating a need for an interdisciplinary common language. Secondly, we analysed the peer-reviewed literature in order to ascertain where marine mammal publications exploring welfare were being published. From 1950 to July 2020, a total of 299 articles featured both marine mammal taxa (one or more) and the word *welfare* in the title, abstract or keywords. This represents just 0.96% of the total peer-reviewed published papers on marine mammal taxa (*n* = 31,221) during the same period. When examining articles published within “Welfare and Ethics” (*n* = 6133) and “Aquatic-focused” (*n* = 139,352) journals, just 1.2% (*n* = 71) and 0.04% (*n* = 57) of articles, respectively, featured the word welfare when examining marine mammals. With the aim of exploring how explicitly including welfare evaluations in marine mammal research and management can benefit conservation outcomes, we framed our workshop and quantitative literature review findings to provide practical solutions to the language, translation and reception issues of this burgeoning cross-disciplinary collaboration.

## 1. Introduction

The multi-disciplinary field of animal welfare science encompasses behaviour, physiology, health and cognition and is improving our understanding of how animals cope with their surrounding environment [1,2,3]. This now well-established scientific discipline emerged in the farm setting, diversified to laboratory and companion species, and is now being rapidly applied to zoo animals [3]. Whilst the application of animal welfare science as a scientific discipline to wild species remains limited, in recent years, our increasing impacts on wild animals and the need for welfare to be considered in conservation have started to be acknowledged [4,5]. When defining animal welfare, scientists have historically been proponents of one of three definitions, where either welfare is determined based on the animal’s health and biological functioning, welfare is based on whether the animal is living a “natural life”, or welfare is dictated by what the animal is feeling [6]. The lattermost “feelings-based” definition has become the most widely-accepted, but actively includes health and natural behaviour since both can impact quality of life [3,7,8,9,10]: “welfare is a dynamic integration of “fitness” and “feelings” (p. 1, [11]). Welfare assessments have also progressed from measuring only the resources available to the animal (“resource-based measures”) and how they might cause suffering to how positive welfare is induced and what indicators the animals show when in such states (“animal-based measures”) [12,13].

As progress in the field of welfare science continues, the potential for measuring affective states in a wide range of contexts is starting to be realised. Most recently, welfare principles are starting to be applied to wild animals to complement conservation efforts, especially in the context of reintroduction programmes [14] and intervention strategies [15,16]. Since welfare concerns an animal’s balance of affective states in response to their surroundings, this is equally applicable to animals in intensively and extensively managed environments, as well as the wild [17,18]. Animal welfare science shares many similarities with conservation biology: both involve multi-disciplinary evaluations of how animals respond and cope/thrive in their environment [15,18,19]. However, the primary difference is that conservation scientists generally consider whole populations in their work, whereas welfare measurement tends to be conducted at the individual level wherever possible [11,16,20]. It has recently been suggested that a more individual-centric approach might aid in addressing certain conservation problems. For example, measuring welfare parameters of individual animals can reveal the impacts of some human activities (e.g., entanglement) on a population or species faster than if long-term data on population-level effects were favoured [19]. Welfare-focused approaches are also thought to reflect societal concerns more accurately in some cases, assisting the public in connecting with the issue [16,21,22] and influencing both social licence and decision-making [20] accordingly. Lastly, the complex and dynamic changes observed in global environments in recent decades require a greater interdisciplinary approach in both research and precautionary and active policy decision-making [21]. For example, it has been argued that relying on abundance data as an indicator of population-level declines would result in the non-detection of the majority of marine mammal species declines [22]. As such, individual approaches may be a crucial addition to contemporaneous monitoring and conservation of populations [19].

The welfare of wild terrestrial animals has been considered for various species and contexts, some only on a theoretical basis [18,19,23] and others involving direct assessments [24]. Common applications have included welfare research into the effects of control programmes on pest species [23,25,26], on species involved in human-wildlife conflict [27], or on methods of killing or darting wild animals for leisure and research [28,29]. The Five Domains model is the most widely used for captive animal welfare evaluations, where collecting data on the first four functional domains, namely nutrition, environment, physical health and behaviour, will inform conclusions on the fifth domain, mental state [10]. Recently, the Five Domains model was adapted into a Ten Stage Protocol for assessing the welfare of individual, non-captive wild animals [24]. Despite these advances in terrestrial wild animal welfare, comparable welfare research on wild marine species appears to have lagged considerably behind. Difficulties in finding, retaining, marking and re-capturing marine mammals in the often-unpredictable marine environment may be one of the major reasons for this, as well as the fact that as terrestrial animals ourselves, we may inherently have more barriers to conceptualising the welfare of marine mammals.

Free-ranging marine mammals face an increasing number of changes to their environment, the vast majority of which are anthropogenic threats [30,31,32], in some cases causing significant population declines and extinctions [19,33]. As with terrestrial animals, contemporary conservation research on marine mammals is increasingly acknowledging these threats, but the difference is that population-level fitness impacts are still seen as the single and most valid measure of success [19,32]. For example, many studies have described the non-lethal disturbance or chronic stress of cetaceans as a result of tourism activities [34,35,36,37,38], but did not refer to impacts on welfare or emotional states. Another example is the recent application of a Population Consequences of Disturbance (PCoD) conceptual framework to marine mammals, where data on individual animals’ behaviour, health and physiology were modelled to understand population effects. However, while the focus remained on loss of fitness and proportion of the population affected, welfare was not mentioned [39].

Despite the novelty of applying animal welfare principles to conservation strategy, some marine mammal conservation projects have started to include welfare considerations. For example, welfare was the central theme of several reviews on the impacts of anthropogenic activities on marine mammals [40,41], and was the focus of a workshop on marine mammal policy and legislation in the UK [42]. There was a Special Issue on animal welfare in a prominent marine mammal journal [43], and a recent comprehensive book on human-induced change and its impacts on marine mammal welfare [30]. A few initiatives are progressing so far as to include welfare science principles and frameworks, such as the International Whaling Commission’s (IWC) workshop on applying the Five Domains model to wild marine mammals [44], which has led to the further adaption of this model in the context of specific threats to marine mammals [31].

Whilst this increasing awareness of wild marine mammal welfare is very encouraging, there remain factors at play that have restricted its adoption in marine conservation, potentially limiting current efforts to comprehensively protect marine mammals [19,40]. This manuscript reports on a two-stage practical and theoretical approach to investigate those limiting factors. Firstly, the current understanding of animal welfare science by cetacean researchers, managers and wider stakeholders was assessed via an international workshop. In the second stage, an analysis of the English language peer-reviewed literature was completed to address two key questions: (1) how often is welfare featured in marine mammal articles, and (2) which types of journal are articles featuring welfare published within? Given the calls for increased cross-disciplinary action in the literature, we hypothesised that current awareness and reporting of wild marine mammal welfare may be limited, and thus the aim of this manuscript was to explore possible underlying mechanisms and solutions to address this.

## 2. Methods

### 2.1. Welfare in the Wild Workshop—Assessing Current Understanding of Welfare Science and Its Application to Marine Mammals

The workshop was hosted by the European Cetacean Society (ECS) during the 32nd conference held in La Spezia, Italy on 6 April 2018. The ECS is an organisation of marine mammal researchers, conservationists, welfarists, veterinarians, industry workers, and many other stakeholders. Whilst animal welfare matters had been discussed at prior conferences and within ethics committee meetings (typically in relation to captive research), animal welfare science (as a scientific discipline) had not been specifically explored by the organisation in terms of its application to cetacean research and conservation. The workshop was open to any attendee of the ECS conference and involved 30 international participants with areas of expertise covering marine mammal conservation, welfare, ecology, behaviour, veterinary medicine, rescue and rehabilitation, policy, and whale-watching.

A 10-question pre- and post-workshop survey was designed to explore participants’ views on animal welfare science both prior and subsequent to the workshop, and to understand whether the workshop itself had altered participant perceptions of the subject. The survey included six open-ended response and a further four 5-point Likert scale [45,46] questions. The surveys were hosted on SurveyMonkey (www.surveymonkey.com; Portland, OR, USA, accessed on 1 March 2018) and distributed one month prior to the workshop and immediately after the workshop. Both surveys each included the same 10 questions (Table S1). In total, 70% of participants (*n* = 21) completed the pre-workshop survey, while 50% (*n* = 15) completed the post-evaluation survey with a third (33%, *n* = 10) completing both the pre- and post-workshop surveys (Table S2). This small sample of paired responses meant that our reporting of the survey results remains descriptive only.

The workshop was structured around four themes (welfare science principles; welfare issues for wild cetaceans; captive cetacean welfare and its applications to the wild; application of welfare science to conservation management), designed to explore the concept and application of welfare science to cetaceans generally, before exploring the specific challenges with wild cetacean welfare assessment and case studies. Each topic was introduced via five-minute explanatory presentations made by each of the co-chairs (IC and KS). In order to conduct participative exercises within each theme, the workshop participants were split into four breakout groups, with each group composed of members with a range of professional backgrounds and career stages. Participants were then led through one participative exercise per theme, designed to consolidate their knowledge and understanding of different cetacean welfare scenarios. Each exercise lasted approximately 45 mins and included reporting back to the wider collective from each breakout group.

### 2.2. Peer-Reviewed Literature Analysis

In order to investigate the frequency and context of current welfare reporting and/or research on marine mammals, we analysed all English language peer-reviewed literature on marine mammal taxa (i.e., articles with any marine mammal taxa in the title, abstract or keywords) published between January 1950 to July 2020. Searches were carried out using Web of Science (all databases) and Google Scholar. To address the prevalence of articles focused on the welfare of marine mammals, we conducted a search for all peer-reviewed journal articles detailing any marine mammal taxa and welfare terms in either their title, abstract or keywords. To capture all marine mammal taxa, we used the search terms: porpoise OR dolphin OR whale OR manatee OR dugong OR “sea otter” OR “polar bear” OR cetacean OR pinniped OR seal OR “sea lion” OR “marine mammal” OR delphinid OR mysticete OR odontocete. To identify welfare articles, we used the search term *welfare*. Additionally, to ensure that we captured all welfare-related articles including those that may be focusing on welfare via physiological indicators, we also included the terms *stress* and *cortisol* [47,48,49,50]. To examine changes in the prevalence of studies focused on marine mammal welfare, we assessed the number of articles published in each decade. To address which journals featured articles detailing marine mammal welfare, nine categories of journal (Veterinary and Medical; Welfare and Ethics; Aquatic; Zoology and Animal Science; Conservation and Ecology; Biological; Environmental; Management, Law, Policy and Economics; and Non-descript) were described based on journal scope as detailed on the website and guidance notes to authors (Table S3).

A direct comparison of the presence of marine mammal welfare articles in Aquatic vs. Welfare and Ethics journals was further assessed. Aquatic journals were identified as such if they had a focus on aquatic environments and species in the journal’s scope. Welfare and Ethics journals included publications that had the words welfare or ethics listed in the journal’s scope (with the exception of human welfare journals, which were eliminated).

## 3. Results

### 3.1. Pre- and Post-Workshop Survey

While small samples of survey respondents precluded statistical analyses being performed, the main findings are reported descriptively here. Overall, the majority of pre-workshop survey respondents (48%, *n* = 10, out of 21) provided definitions of welfare that aligned with the biological functioning definition (i.e., welfare is determined by the animal’s physical fitness), followed by 33% (*n* = 7, out of 21) whose definitions aligned with an animal’s affective state (Table S2).

Prior to the workshop, there was a relatively even distribution of responses to the 5-point Likert scale question ‘When discussing the welfare of a wild animal or a captive animal, do you consider it the same concept?” with 33% of respondents (*n* = 7, out of 21) choosing to “Agree” or “Strongly agree”, and 38% (*n* = 8, out of 21) neither agreeing nor disagreeing. The post-workshop survey answers suggested more certainty, with 79% of respondents (*n* = 10, out of 15) agreeing with the question’s statement (Table S2).

### 3.2. Literature Analysis of Marine Mammal Welfare Articles

Of all English language peer-reviewed journal articles published from 1950 to July 2020 detailing any marine mammal taxa (*n* = 31,221), only 0.96% (*n* = 299) of articles featured the term welfare, with 1.8% (*n* = 563) detailing stress and 0.76% (*n* = 236) featuring cortisol. Of all articles published in Aquatic journals (*n* = 42 journals) between 1950 and July 2020 (*n* = 139,352), there were 0.04% featuring welfare (*n* = 57), 0.08% that described stress (*n* = 117), and 0.03% that detailed cortisol (*n* = 39) in the title, abstract or keywords. Of all articles (*n* = 6133) published in 17 Welfare and Ethics journals between 1950 and July 2020, only 1.2% of articles (*n* = 71) described marine mammal taxa in the title, abstract or keywords.

If we consider the above results as a percentage of all journal articles detailing any marine mammal taxa (*n* = 31,221), just 0.20% (*n* = 62) of all marine mammal welfare articles were published in Welfare and Ethics journals, while articles referencing welfare and published in Aquatic journals comprised just 0.18% (*n* = 57) of all marine mammal articles published within the same time span.

**Figure 1:**
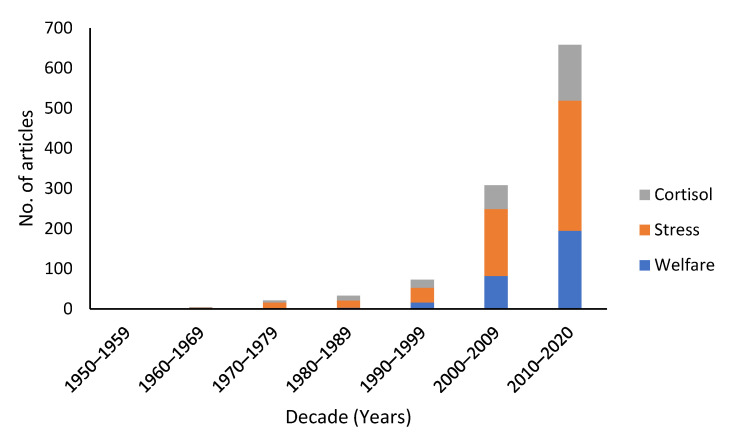
Number of published articles that focused on marine mammal welfare, by including the terms welfare, stress or cortisol, in the title, abstract or keywords between 1950 and 2020.

Chronological examination revealed a substantial increase in the number of articles published decade by decade that were focused on marine mammal welfare (in any journal) when including any of the following terms: welfare, stress and cortisol (Figure 1). A four-fold increase in the total number of articles published between the decades of 1990–1999 (*n* = 73) and 2000–2009 (*n* = 308) was noted. This increase was further evident in the decade of 2010–2019, where it further doubled (*n* = 658).

## 4. Discussion

Our results from the literature review, supported by the descriptive findings from the workshop, suggest that there are varying views about both animal welfare and animal welfare science among cetacean scientists and other stakeholders. In the peer-reviewed literature, only a small fraction of articles on marine mammal taxa focused on their welfare, either when the exact term was used or when it was inferred via the use of the terms stress and cortisol. We also found discrepancies regarding publishing location; when examining all marine mammal articles published within Welfare and Ethics and Aquatic journals since 1950, only 1.2% and 0.04%, respectively, featured the word welfare.

### 4.1. Welfare in the Wild Workshop

The workshop and discussions generated within it highlighted varying views as well as some misconceptions about animal welfare among cetacean scientists and stakeholders. This also aligns with what has previously been reported for welfare in relation to terrestrial animals [11,14]. Although we can only descriptively report on the workshop findings due to the small sample size, we conservatively note that the majority of participants who responded to the pre-workshop survey defined welfare using terms that were most aligned with the biological functioning definition [51]. This finding is supported by other studies that found that terrestrial animal researchers tended to perceive animal “fitness” as the dominant component of good welfare [11,16]. Furthermore, it has been suggested that stakeholders are likely to subscribe to definitions of welfare that most align with the goals of their discipline [11], the industry they work in [52], or their personal ethical values [51].

Over the course of the workshop, we anecdotally and conservatively observed an increased acceptance of the possibility of measuring welfare of wild marine mammals, as more respondents post-workshop agreed that welfare as a concept could be applied in both wild and captive settings. An enhanced understanding of welfare-related topics common to both conservation and welfare science may have been key to this outcome, and other authors have proposed specific topics that might act as a “common language”, including stress, distress and suffering [11], which were all a focus of the workshop. In all cases, a foundational, common understanding of animal welfare principles is crucial to any cross-disciplinary application to conservation [16,53].

### 4.2. Literature Analysis of Marine Mammal Welfare Papers

Of all articles published on marine mammal taxa between 1950 and mid-2020, less than 1% focused on welfare (with similar results also reported for the words stress and cortisol). This demonstrates that either (1) studies of welfare are currently limited in marine mammals, confirming authors’ previous suggestions that this research is likely lagging behind terrestrial efforts [19,40] and/or (2) welfare is included within marine mammal studies but not considered as an important or priority contribution to the study or its objectives (based on its absence from the title, abstract or keywords). In terms of absolute numbers of published articles on welfare of marine vs. terrestrial animals, our analysis revealed that the numbers are currently in the low hundreds, whilst a recent study on terrestrial animal literature found that the total number of welfare articles was nearing 8000 by 2017 (noting methodological differences in search terms) [54]. Comparing our results with Freire and Nicol’s analysis also confirms that the exponential increase in welfare articles concerning marine mammals (Figure 1) seemed to occur at least two decades later than for terrestrial taxa [54]. Although our literature analysis revealed an increasing trend in welfare-focused published studies, it also indicated that as a percentage of total articles published since 1950, a higher percentage of articles focused on marine mammal welfare feature in Welfare and Ethics as opposed to in Aquatic journals (1.2% vs. 0.04%). This could indicate one or more of the following: that Welfare and Ethics journals are currently more likely to publish marine mammal welfare articles, they are more targeted by marine mammal welfare authors than Aquatic journals, and/or more welfare scientists are studying marine mammals than the inverse.

The paucity of marine mammal welfare literature, and the suggested trend that any existing research is more commonly published in welfare journals than other areas, indicates that welfare science principles have still not been effectively translated to, or received by, those working with and publishing on marine mammals. For example, when reviewing journal websites for scope and author guidance, welfare remained notably absent from almost all current conservation journals examined. Furthermore, in cases where manuscript submission requires authors to select themes, categories or keywords via dropdown lists, no options appeared for either welfare or conservation welfare on any of the conservation-based portals examined. Coupled with our workshop findings and literature analysis results, these insights support previous conclusions regarding the lack of synthesis between welfare science and conservation biology [11,24,30,53,55,56,57,58]. To address this, the necessary next steps should be practical in nature and must expand the discussion beyond terrestrial examples. In the remainder of this paper, we suggest solutions to the fundamental language, translation and reception issues highlighted in the literature and by our two studies, with a focus on the context of marine mammal conservation.

### 4.3. The 3 Challenges of Cross-Disciplinary Collaboration: Language, Translation and Reception

#### 4.3.1. The Language Pertaining to Wild Marine Mammal Welfare

Whenever two or more disciplines convene, a common language must be established since words and terms often have varied meanings across different fields [11,59]. Welfare itself is actually a concept whose meaning varies across disciplines, and understanding of the term as central to the scientific discipline animal welfare science requires knowledge of the paradigms contained within it [59]. This is supported by findings from the workshop described in this manuscript, where researchers seemed to define welfare depending on their area of expertise, and the majority chose a biological functioning or “fitness” definition. As discussed in a recent conservation welfare perspective [11], instead of immediately insisting on a common definition directly from welfare science (i.e., welfare concerns how the animal’s experiences and “feelings”), it may be more constructive to first introduce the idea that welfare is both about “feelings” as well as “fitness”, before later introducing the concept that fitness is only relevant to welfare if it impacts the animal’s emotional state. “Naturalness” is another concept which in the past has been strongly linked to welfare, but now, similar to fitness, is considered to only impact welfare if it affects the animal’s emotional state. Nevertheless, a recent review found that while existing research failed to provide a link between the commonly used criterion of “natural behaviour” and welfare, this idea may continue to be favoured due to different teleological conceptions of welfare itself, i.e., that only through performing natural behaviour can the animal demonstrate good welfare [60]. This alternate definition of welfare is likely to be held in some form among cetacean biologists given that 20% of our workshop survey respondents articulated this concept. Therefore, those aiming to investigate welfare impacts on conservation outcomes might consider holding participative discussions or workshops concerning welfare definitions.

Outlining the terms relating to conducting welfare evaluations, such as measures, assessments, and validation of measures, should also be part of the initial knowledge-sharing process for marine mammal welfare projects. Concepts that are commonly studied in both fields should be identified, both in order to highlight areas for collaboration but also to ensure that they are understood in the same manner [59]. For example, between marine mammal and welfare science, some of the key shared concepts to bring to collaborating partners’ attention would be the topics of stress, distress and perhaps suffering (see [11] for an explanation of their differential use between disciplines), but also personality, culture, and sociality [61].

Similar to the reasoning for including welfare principles in conservation, it has been demonstrated that expanding our view from population-level-only epidemiological metrics to including individual or group behaviours regarding sociality and culture can also benefit conservation efforts [19,61,62] Many marine mammals are gregarious and have cultural behaviours that they adopt from each other through social learning [33,61,62]. Any future considerations of marine mammal welfare should integrate concepts such as sociality and culture, both as a measure when evaluating welfare, and as a tool during intervention efforts [62]. Stranding events of wild marine mammals are a key example of how social behaviour could drastically impact welfare. Mass strandings predominantly involve pelagic odontocete species (e.g., pilot whales, sperm whales), where it is hypothesised that strong social cohesion is a causal factor in the stranding event, with most individuals diagnosed as otherwise outwardly healthy [44,63,64]. Strandings have been the source of much stress-related data, given that the animals are generally experiencing acute to chronic poor welfare states (i.e., from the stress of the stranding event to the longer-term physiological stressors which may have caused the stranding). Notable progress relevant to measuring welfare includes the use of scanning electron microscopy observation to determine when noise exposure occurred [65], and on-the-beach blood profile tests to inform triage decisions [66]. However, the literature review revealed that just 1.7% (*n* = 5) of articles on marine mammal welfare focused on strandings. Other wild marine mammal contexts involving direct human actions (including entanglement, whale-watching, ship strike and hunting) are also starting to be studied in a welfare-centric light, which in turn is revealing how these activities are impacting individual animals [40,41,67,68,69]. Of these, hunting is particularly well represented in the marine mammal welfare literature (17.7%, *n* = 53), compared to boat disturbance (11.4%, *n* = 34; including all maritime traffic inclusive of whale-watching), and entanglement (5%, *n* = 15). Another emerging area of marine mammal welfare relates to scientific research itself, particularly in the use of tags and other invasive mark-recapture tools, with 5.7% (*n* = 17) of articles in the literature search specifically referencing welfare in relation to these practices. It is well-accepted that the benefits of the mark-recapture research should be weighed “against the method’s associated harms to the individual animal” (p. 55, [70]), and those direct and indirect harms can only be effectively assessed using an objective and comprehensive welfare framework. This particularly complex intersection of welfare science and ethical viewpoints [71,72] is where the acceptability of welfare outcomes may differ depending upon the ethical standpoint [73].

In fact, two schools of thought have developed in regard to the interactions between ethics, welfare and conservation policies: “compassionate conservation” and “conservation welfare” [11,73]. The differences between them are important to reinforce, since confusion between the two may limit the uptake of welfare principles by conservationists [11]. These two fields are in fact underpinned by different ethical beliefs, where compassionate conservation insists that conservation should “do no harm”, and conservation welfare aims to improve welfare for as many animals affected by conservation interventions as possible [73]. An example of where these approaches tend to disagree in terrestrial systems is the control of introduced species [74], where compassionate conservationists would advocate for non-invasive and non-lethal methods to control predator species, but conservation welfarists may favour these methods if they resulted in better welfare outcomes for large populations of prey species. The differences between these fields, and indeed the potential misconceptions about welfare science by conservation researchers, are comparable to the conflation with the animal rights movement that welfare science suffered earlier in its genesis [75]. Clear communication of animal welfare science principles and its lack of an ethical position, and increased collective thinking opportunities [21], should be considered in order to bridge the knowledge gap that this study has revealed.

#### 4.3.2. The Translation of Welfare Science Information to Marine Mammal Conservation

Assuming that a common language regarding welfare science is understood and agreed upon for wild marine mammal welfare, the next issue is the “translation” of this field, which describes the movement of information from one discipline to another, including technical terms and methods whilst taking into account the boundaries of discipline-specific knowledge [59]. Published welfare science studies on marine mammals to date have either applied welfare models and/or assessed welfare in captive environments. Welfare models such as the Five Freedoms [76] or the Five Domains [10] are frameworks designed to be applied to any animal (and largely in any context) that help users to consider the wide range of welfare indicators that might be relevant. The Five Domains framework has been adapted to measure the welfare of wild animals in general [24], and just recently, specifically applied to cetaceans in the format of a Welfare Assessment Tool for Wild Cetaceans (WATWC) [31]. The WATWC was developed using an iterative process and a wide range of experts and stakeholders, and the scoring framework has undergone pilot testing to arrive at a refined version, for which there was high assessor agreement. While only a first step towards the measurement of wild cetacean welfare, importantly the authors showed that welfare science models can be successfully applied to wild marine mammals [31]. The tool has already been adapted and trialled at a marine mammal welfare workshop held by Whale and Dolphin Conservation and the Wild Animal Welfare Committee in the UK in 2019 to assess the welfare impacts of bycatch and wind farm construction on harbour porpoises (*Phocoena phocoena*) [42].

Though there is an increasing realisation of the need for welfare to be considered in conservation [4,16,21], the application of welfare science to wild species has been focused predominantly on captive settings (i.e., zoos, aquaria, rescue centres and sanctuaries) [11,24]. Indeed, this bias was demonstrated by the fact that 26.4% (*n* = 79) of marine mammal welfare articles from the literature review focused on captive animals. Studies on marine mammal welfare in captivity have been increasing over the last 10 years [77,78], and using dolphins as an example, there is now existing research on positive welfare indicators [79,80,81,82,83,84], negative welfare indicators [82,85,86,87], and a comprehensive assessment framework [88]. Physiological and health-related welfare parameters such as stress hormones [86,89,90], blood profiles [91,92], and pulmonary function [93] have also been examined in captive animals. The controlled environment of the captive setting has also aided in the development of certain hydrophone-video arrays aiming to match cetacean vocalisations to behaviours, thereby decoding their meaning, which would be a critical step in evaluating cetacean welfare [94,95]. However, much is still unknown about the utility of captive welfare findings to wild marine mammals. The feasibility of data collection will of course be a limiting factor, where welfare indicators developed for captive animals such as weight measurements, blood profiles and cognitive bias testing that may be easily carried out on trained animals are likely to be difficult to undertake in the wild. The applicability of captive animal physiological reference ranges to wild marine mammals remains an ongoing discussion [66]. Some assert that captive research can successfully be adapted to aid wild marine mammal conservation [57,89,95,96,97], while others advocate that welfare in captivity is compromised to the point that wild-captive comparisons of behaviour, physiology or cognition are invalid [98]. For larger species such as baleen whales and other species not kept in captivity, there is very limited research from a few longer-term rehabilitation efforts that can be used to evaluate its applicability [99,100]. Nevertheless, the study and management of wild animals in zoos embodies a current synergy between the conservation and animal welfare fields [11], including for marine mammals.

A recent collaboration linking wild and captive marine mammal welfare was established between the International Union for Conservation of Nature (IUCN), the Species Survival Commission, the National Marine Mammal Foundation, other IUCN groups, NGOs, and several zoos that display cetaceans. These diverse stakeholders held a workshop on “Ex Situ Options for Cetacean Conservation”, where strategies were discussed for critically endangered cetacean populations for whom some form of captive breeding and reintroduction was considered to mitigate continued population decline. Wild cetacean welfare was mentioned several times in the proceedings and appears to be a central theme of any intervention proposals [101]. It should be noted that there are ethical implications of such interventions since they may result in compromised welfare or even death for a few individuals. Until now, bringing animals into captivity or into a different environment to their home range to breed and then reintroduce has only occurred when many other conservation efforts have been attempted and failed, such as for the Yangzte finless porpoise (*Neophocaena asiaeorientalis asiaeorientalis*), whose population has doubled since they entered their ox-bow lake “semi-natural” environment, but who now face a risk of in-breeding [102,103]. There are clearly further, complex discussions which are needed to improve efforts to prevent such measures being required and, only as a last resort, establish the point in a population’s decline when ex situ breeding programs should be commenced [101,104].

A key aspect of translating information within cross-disciplinary collaborations lies in identifying the limitations of the research and knowledge being shared [59]. In terms of translating knowledge on welfare models, a ubiquitous limitation is that any evaluation of marine mammal welfare will only be a best estimate, as we can never know exactly how the animal is feeling [1]. Furthermore, due to the complexities of finding free-ranging marine mammals and observing the same individuals over long time periods, welfare assessments may only provide a snapshot of individual welfare at that time [24], so measures of behavioural interactions and environmental variables that require a time series may not be applicable. The translation of welfare science principles into wild marine mammal conservation may also face some social and cultural limitations [59], given the ethical debate surrounding captive marine mammals [57]. Fostering open discussions to enable consensus-building and develop clear Terms of Reference documents might be a productive first step in the process of addressing the varying views, definitions and language currently used, and may help researchers to find common ground and pave the way for translating the applicable information.

A final aspect to consider in relation to the translation of information is which organisations might be driving this exchange. Those academic and research institutes that already have departments or research staff in both welfare science and conservation biology should be encouraged to foster these much-needed transdisciplinary collaborations. International organisations are also capable of igniting this work, where current examples include the IWC’s series of workshops exploring the welfare impacts of anthropogenic threats to cetaceans [31,105], and the IUCN’s ex situ options for cetacean conservation working group [101]. In direct reference to integrating welfare science with other disciplines, it has been asserted that organisations “can encourage interactions by providing infrastructure support and reducing artificial structural boundaries between disciplines” (p. 46, [65]). For example, funding bodies need to include panels that are not rigidly discipline-based and are able to assess complex interdisciplinary applications [59]. Looking forward, undergraduate marine mammal conservation programmes should consider integrating welfare science modules into their courses, thereby encouraging future post-graduate and early career research in matters of wild marine mammal welfare.

#### 4.3.3. The Reception of Welfare Information by Marine Mammal Scientists

After the language and translation issues in a collaboration have been addressed, the reception of welfare information should be considered further [59]. In regard to marine mammal welfare, this concerns how and where such research is published and communicated, and as our literature analysis demonstrates, currently this is more likely in the welfare science journals. To address this, marine mammal science and conservation journals could consider expanding their scope to include terms and topics relevant to welfare, perhaps initially by proposing special topics. This was trialled by *Aquatic Mammals* in 2018 [43], representing a first successful effort to generate interdisciplinary synergies between welfare and marine mammal science. Further workshops and seminars similar to that presented here that address the nexus between conservation biology and animal welfare should be organised, with full conferences on marine mammal welfare considered to encourage cross-disciplinary collaborations. Such efforts need to be accessible (geographically, socially and economically) to both welfare and conservation scientists working with marine mammals, and secondarily to wider stakeholders, a feat perhaps more feasible with increased virtual conferencing following the COVID-19 pandemic [106]. Societies and organisations are encouraged to consider animal welfare committees whose role extends beyond matters of captivity and ethics, as is the current status quo. Given that collaborations in today’s world benefit from instantaneous communication between stakeholders, as well as real-time engagement with the public through channels such as social media, an information-sharing platform could be established with regard to the study of marine mammal welfare with the aim of connecting all researchers currently working on relevant aspects and allowing organisation and dissemination of research.

Of course, there are many other challenges hindering research into marine mammal welfare, and its potential impact on conservation strategy. Funding for such collaborations is a key issue: currently, the majority of funding bodies offer either grants for welfare projects with domestic animals or fund conservation projects using traditional population-level methods, with cross-disciplinary projects falling outside the scope of both. Through open communication and increasing involvement in workshops and early-stage discussions, it is hoped that funding bodies see the value of cross-disciplinary research by providing support for projects aiming to integrate welfare science principles into marine mammal conservation. By “intentionally managing the collaboration” [21], i.e., proactively addressing the language, translation and reception of welfare science information in marine mammal conservation, the emerging discipline is more likely to be successful and sustainable. Table 1 summarises the suggested objectives described to encourage successful collaborations between welfare science and biology as it pertains to marine mammals, with suggestive methods to achieve this.

## 5. Conclusions

Marine mammal conservationists are tackling increasing anthropogenic threats that impact all aspects of the animals’ lives. While there are encouraging signals of increasing cross-disciplinary thinking in the welfare science and terrestrial conservation space, there has to date been limited uptake in marine mammal conservation. This study investigates those limiting factors, with results suggesting that a normative environment is not yet established for wild marine mammal welfare research (i.e., one that provides funding, infrastructure, and adequate cross-disciplinary support culture). We identify how these language, translation and reception issues can be addressed and suggest practical next steps for how welfare science principles might be integrated into marine mammal conservation going forward. Our work provides a framework of actionable objectives for individual and organisational collaborators who are motivated to apply new, cross-disciplinary thinking and approaches to mitigate the comprehensive and ever-increasing threats faced by wild marine mammals.

## Figures and Tables

**Table 1:** Suggested objectives and methods for addressing the language, translation and reception issues relating to applying welfare science principles to marine mammal conservation.

	Objective	Methods
**Language** Ensuring terms and concepts have common meaning	1. Establish a common understanding of “welfare”, including affective states, emotions, and welfare models	Workshops, seminars or conferences involving welfare and conservation scientists, develop a shared ‘Terms of Reference’ (ToR) document. Share ToR document with all stakeholders (researchers, journals, NGOs, governmental organisations, consultants) Collaborate with those already working at the terrestrial conservation welfare interface to share experiences, especially regarding differing opinions on welfare definitions, e.g., natural behaviour. The lack of ethical position in welfare science should be emphasised to reduce misalignment with animal rights.
	2. Highlight areas where shared concepts exist between marine mammal conservation and welfare science, such as stress, distress, suffering, personality, culture, and sociality	Review existing literature in both welfare science and marine mammal science that uses the same welfare-related terms. Specific collaboration between those working in these areas in both fields, open discussion of results and interpretation. Engage those already working on these topics in terrestrial conservation welfare to learn how they overcame any language issues.
	3. Identify welfare tools relevant to marine mammal conservation: measures, assessments, validation of measures	Series of workshops, seminars or conferences involving welfare and conservation scientists, develop a shared ‘Terms of Reference’ document. Suggested start: discussions and theoretical applications of the Nicol et al. (2020) adapted Five Domains framework for wild cetaceans [31].
**Translation** Enabling the movement of information from one discipline to another	4. Collaborations between welfare scientists and marine mammal researchers: share knowledge between these two fields, explore whether any experience or resources from any type of captive setting is applicable	Conferences, workshops, and seminars where welfare scientists and conservation researchers can meet and collaborate, especially since there are several existing research groups that are geographically close. Encourage/lobby funding bodies to increase awareness and support for projects aiming to foster cross disciplinary collaborations Researchers can also look outside the box for funding sources for such cross-disciplinary, international, and culturally significant collaborations.
	5. Universities or organisations that already have research interests in both welfare science and marine mammal biology should take the lead in facilitating information exchange	Universities and organisations could support through funding, support culture, and infrastructure. Increased dialogue should be encouraged, and collaborations incentivised. Welfare science principles should start being taught in undergraduate marine mammal biology and conservation programs.
**Reception** Ensuring the effective dissemination of results	6. Inter-disciplinary conferences to allow translation of information and communication of results	Workshops, seminars, and conferences that are accessible to both welfare and conservation scientists. Animal welfare committees should be established in marine mammal societies and organisations to manage any welfare topics in conservation research and management.
	7. Work with journals and Universities to encourage commitment to inter-disciplinary papers	Special issues on wild marine mammal welfare. Inclusion of welfare and conservation welfare within journal scope and keywords. Encouragement of new, cross-disciplinary journals.
	8. Platform/common database set up for information, communication, and coordination	Terms of Reference documents, virtual learning resources, conference announcements, calls for papers, grants, and findings could all be shared on such a platform in real-time. Harness potential of social media communication.

## Data Availability

The data presented in this study are available in the Supplementary Materials.

## Supplementary Materials

The following are available online at https://www.mdpi.com/article/10.3390/ani11061596/s1, Table S1: The 10 questions asked within the pre- and post-workshop surveys; Table S2: Results of selected survey questions described in the main body of the manuscript; Table S3: Categorisation of journals into types depending on scope.
