# Supplementary material for: Increasing the Awareness of Animal Welfare Science in Marine Mammal Conservation: Addressing Language, Translation and Reception Issues
Source: Animals (Basel). 2021 May 28;11(6):1596. doi: 10.3390/ani11061596 (PMC8230206; doi:10.3390/ani11061596)
Supplement: Supplementary file 1 [file animals-11-01596-s001.zip › animals-1218569-supplementary.pdf]

Supplementary Materials

Increasing the Awareness of Animal Welfare Science in Marine Mammal Conservation: Addressing Language, Translation and Reception Issues

Isabella L. K. Clegg, Rebecca M. Boys, Karen A. Stockin

Table S1. The 10 questions asked within the Pre-workshop and Post-workshop surveys.

Category	Question type	Question	Pre- workshop survey	Post- workshop survey
Participant details	Open-ended	1. Please list the principal organisation or institution you are associated with, your current occupation, and where your area of expertise or experience lies.		
Animal welfare	Open-ended	2. How would you define animal welfare?		
	Open-ended	3. How do you think animal welfare might be measured?		
	5 point Likert scale: Strongly Disagree to Strongly Agree	4. When discussing the welfare of a wild animal or a captive animal, do you consider it same concept? Please provide brief reasons for your answer.		
	5 point Likert scale: Strongly Disagree to Strongly Agree	5. Should welfare concerns of individuals outweigh conservation efforts for a population?		
Wild cetacean welfare	5 point Likert scale: Strongly Disagree to Strongly Agree	6. Is measuring a wild cetacean's welfare relevant to conservation efforts? Please provide brief reasons for your answer.		
	Open-ended	7. What are the three types of circumstances where you believe wild cetaceans are experiencing the poorest welfare?		
	5 point Likert scale: Strongly Disagree to Strongly Agree	8. Do you think animal welfare measurement might be useful in your work? Please provide brief reasons for your answer.		
	Open-ended	9. What do you perceive to be the biggest challenge of assessing welfare in wild cetacea?		
	Open-ended	10. Are you familiar with the concept of 'Conservation Welfare': how would you imagine it to be defined?		
Workshop outcomes	Open-ended	11. What are the 3 main things you learnt from workshop?		

Table S2. Results of selected survey questions referred to in the main body of the manuscript.

Question	Which survey (and how many respondents)?	Any post-response categorisation of answers?	Number of responses				
2. How would you define animal welfare?	Pre-workshop survey (*n* = 21)	Yes: categorised into the three definition types of animal welfare (feelings-based, natural-living, biological functioning) as per Fraser (2009).[1]	Feelings-based definitions		Natural-living definitions		Biological functioning definitions
			7		4		10
4. When discussing the welfare of a wild animal or a captive animal, do you consider it the same concept?	Pre-workshop survey (*n* = 21)	No	Strongly Agree	Agree	Don't know	Disagree	Strongly disagree
			2	5	8	5	1
	Post-workshop survey (*n* = 15)	No	3	7	2	2	0

[1] Fraser, D. Assessing animal welfare: different philosophies, different scientific approaches. *Zoo Biology* **2009**, *28*, 507–518.

Table S3. Categorisation of journals into types depending on scope.

**Veterinary and Medical** (scope: veterinary, disease, anatomy, parasitology, clinical, immunology, pathology)	**Non-descript** (scope: broader science)	**Environmental** (Scope: Toxicology, Physical sciences, Environment)	**Welfare and Ethics** (Scope: Welfare, Ethics)	**Aquatic** (Scope: Marine, Freshwater, Ocean, Coastal, Marine mammals, Fisheries, Sea)	**Biological** (Scope: Physiology, Behaviour, Genetics, Aging)	**Zoology and Animal science** (Scope: Animal, Zoo, Zoology)	**Conservation and Ecology** (Scope: Conservation, Ecological, Wildlife)	**Management, Law, Policy and Economics**
Acta Endocrinologica	Behavior Research Methods Instruments and Computers	Analytical and Bioanalytical Chemistry	Alternatives to Animal Experimentation	Aquatic Conservation-Marine and Freshwater Ecosystems	Annual Meeting of the Society for Integrative and Comparative Biology	Acta Ethologica	9th International Symposium: Environmental Concerns in Rights-of-Way Management, Portland, Oregon, USA, 27-30 September, 2009	Adelaide law review
Acta Parasitologica	Frontiers in Robotics and AI	Annals of the American Association of Geographers	Alternatives to Animal Testing and Experimentation	Aquatic Mammals	27th Congress of the European Society of Comparative Biochemistry and Physiology	Acta Theriologica Sinica	Ambio	Animals and Tourism: Understanding Diverse Relationships
Acta Veterinaria Scandinavica	Green Arguments and Local Subsistence	Aquatic Toxicology	Alternatives to Laboratory Animals	Benthic Habitats and the Effects of Fishing	Acta Physiologica Scandinavica	Animal Biotelemetry	Antarctic Science	Annals of Leisure Research
Advances in Experimental Medicine and Biology	International Seminars on Nuclear War and Planetary Emergencies	Archives of Environmental Contamination and Toxicology	Animal Sentience	Bulletin of Marine Science	Aging Cell	Animal Science Journal	Arctic	Asian Journal of WTO and International Health Law and Policy
American Association of Zoo Veterinarians. Annual proceedings	Internet Research	Arctic Science	Animal Welfare	California Fish and Game	American Journal of Physiology-Regulatory Integrative and Comparative Physiology	Animals	Biharean Biologist	Coastal Management
American Journal of Physiology-Endocrinology and Metabolism	Journal of Materials Engineering and Performance	Biorheology	Animal Welfare in a Changing World	Canadian Journal of Fisheries and Aquatic Sciences	Anatomical Record-Advances in Integrative	Annales Zoologici Fennici	Biological Conservation	Current Issues in Tourism

					Anatomy and Evolutionary Biology			
American Journal of Veterinary Research	Journal of the Acoustical Society of America	Chemosphere	Animal Welfare, Science, Ethics and Law Veterinary Association	Chinese Journal of Oceanology and Limnology	Archives of Oral Biology	Applied Animal Behavioural Science	Biology and Environment- Proceedings of the Royal Irish Academy	Ecological Economics
Anatomia Histologia Embryologia- Journal of Veterinary Medicine Series C	Journal of Visualized Experiments	Comparative biochemistry and Physiology C-Toxicology and Pharmacology	International Journal for the Study of Animal Problems	Ciencias Marinas	Australian Journal of Biological Sciences	Behavioural Ecology and Sociobiology	Bulletin Southern California Academy of Sciences	Environment International
Andrology	MethodsX	Conference on Toxicology and Risk Assessment Approaches for the 21st Century	Journal of Animal Ethics	Deep-Sea Research Part I- Oceanographic Research Papers	Biochemical Genetics	Behavioural Processes	Conservation Biology	Environmental and Resource Economics
Annali della Facolta di Medicina Veterinaria, Universita di Parma	Nature	Ecotoxicology	Journal of Applied Animal Welfare Science	Effects of Noise on Aquatic Life II	Biotemas	Bio-mechanisms of Swimming and Flying: Fluid Dynamics, Biomimetic Robots and Sports Science	Department of the Environment; South Ruislip; UK	Global Trade and Customs Journal
Anthrozoos	Nature Communications	Ecotoxicology and Environmental Safety	Proceedings of the 10th Annual BIAZA Research Symposium, Hull, UK, 15-16 July 2008.	Fish Physiology and Biochemistry	BMC Evolutionary Biology	Cahiers Agricultures	Ecological Applications	Human and Ecological Risk Assessment
Australian Veterinary Association	New Scientist	Environmental Health Perspectives - International Workshop on Ecological Relevance of Chemical-Induced Endocrine Disruption in Wildlife	Social Welfare Interdisciplinary Approach	Fisheries Oceanography	BMC Genomics	Canadian Journal of Zoology	Ecological Indicators	Interfaces
Australian Veterinary Journal	Occupational Therapy International	Environmental Physiology and Biochemistry	Tourism and Animal Welfare	Fisheries Research	BMC Research Notes	Ethology	Ecological Modelling	International and Comparative Law Quarterly
Behavioural Brain Research	Physica A- Statistical Mechanics and its Applications	Environmental Pollution	Universities Federation for Animal Welfare	Fisheries Science	Comparative Biochemistry and Physiology A- Molecular and Integrative Physiology	First Annual Crissey Zoological Nutrition Symposium, Raleigh, North Carolina, USA, 12-13 December, 2003	Ecology	International Journal of Tourism Research
Berliner und Munchener Tierarztliche Wochenschrift	Plos One	Environmental Research	Whale Welfare and Ethics Workshop	Frontiers in Marine Science	Comparative Biochemistry and Physiology B- Biochemistry and Molecular Biology	Integrative Zoology	Ecology and Evolution	Issledovanija po Slavjanskim Jazykam
Biological Psychiatry	Proceedings of the National Academy of Sciences of the	Environmental Science and Technology	World Animal Protection	Hydrobiologica	Comparative Biochemistry and Physiology D-Genomics	International Zoo Yearbook	Ecosphere	Journal of Economic Animal

	USA				and Proteomics			
BMC Veterinary Research	Royal Society Open Science	Environmental Toxicology and Chemistry	World Society for the Protection of Animals; London; UK	ICES Journal of Marine Science	Conservation Physiology	Journal of Evolutionary Biochemistry and Physiology	Endangered Species Research	Journal of Ecotourism
Brain Behaviour and Immunity	Sains Malaysiana	Environmental Toxicology and Pharmacology		Journal of Aquatic Ecosystem Stress and Recovery	Current Biology	Journal of Experimental Biology	European Journal of Wildlife Research	Journal of Environmental Economics and Management
Brazilian Journal of Veterinary Research and Animal Science	Science	Folia Pharmacologica Japonica		Journal of Cetacean Research Management	Cytogenetics and Cell Genetics	Journal of Experimental Zoology Part A-Ecological and Integrative Physiology	Evolutionary Applications	Journal of Environmental Protection and Ecology
Canadian Journal of Comparative Medicine	Science Advances	Gulf and Caribbean Research		Journal of Dalian Fisheries	Evolutionary Bioinformatics	Journal of Experimental Zoology Part A-Ecological Genetics and Physiology	Functional Ecology	Journal of International Wildlife Law and Policy
Canadian Veterinary Journal	Scientific Reports	Israel Journal of Chemistry		Journal of Experimental Marine Biology and Ecology	FASEB Journal	Journal of Mammalogy	Global Change Biology	Journal of Sustainable Development
Commonwealth Veterinary Association	Sustainability	Journal of Toxicology and Environmental Health- Part A Current Issues	Journal of Marine Animals and their Ecology	FEMS Microbiology Ecology	Journal of Zoo and Aquarium Research	Human-Wildlife Interactions	Journal of Sustainable Tourism	Commonwealth Veterinary Association
Developmental and Comparative Immunology	Symmetry-Basel	Journal of Turbulence		Journal of MBA UK	Free Radical Research	Journal of Zoological Systematics and Evolutionary Research	Journal of Threatened Taxa	Korea International Law Review
Diseases of Aquatic Organisms	Transactions of the Royal Society of South Africa	Natturufraeding urinn		Journal of the Fisheries Research Board of Canada	Frontiers in Physiology	Journal of Zoology	Journal of Wetlands Ecology	Korean Journal of International Economic Law
Ecohealth	Water Science and Technology	Neurotoxicolog y and Teratology		Marine and Freshwater Behaviour and Physiology	G3-Genes, Genomes, Genetics	Los Angeles County Mus Contrih Sci	Journal of Wildlife Management	Marine Wildlife and Tourism Management: Insights from the Natural and Social Sciences
Free Radical Biology and Medicine		Polar Record		Marine Biology	Hormones and Behaviour	Mammal Research	Media Konservasi	Review of Economics and Statistics
Frontiers in Endocrinology		Polar Research		Marine Ecology Progress Series	Human Physiology	Mammal Review	Memoranda Societatis pro Fauna et Flora Fennica	Social and Cultural Geography
Frontiers in Immunology		Rapid Communication s in Mass Spectrometry		Marine Environmental Research	Integrative and Comparative Biology	Physiological and Biochemical Zoology	Methods in Ecology and Evolution	Social and Legal Studies
Frontiers in Psychology		Science of the Total Environment		Marine Mammal Science	Integrative and Comparative Physiology	Physiological Zoology	National Geographic Research	Tourism and Hospitality Management
Frontiers in Veterinary Science		Toxicology and Applied Pharmacology		Marine Policy	Journal of Applied Microbiology	Proceedings of the 5th Annual Symposium on Zoo Research, Marwell Zoological Park, Winchester, UK, 7-8th July 2003.	Nature Conservation Bulgaria	Tourism in Marine Environments
General and Comparative		Toxicology and Environmental		Marine Pollution Bulletin	Journal of Applied	Proceedings of the Zoological	Open Conservation	Tourism Management

Endocrinology	Chemistry			Physiology	Society of London	Biology Journal	
Indian Journal of Virology	Toxicology In Vitro		Ocean and Coastal Management	Journal of Biological Chemistry	Reproductive Sciences in Animal Conservation	Raffles Bulletin of Zoology	Tourist Destination Governance: Practice, Theory and Issues
Israel Journal of Veterinary Medicine	Ukrainskii Biokhimicheskii Zhurnal		Oceans 2006 Conference	Journal of Comparative Physiology A-Neuroethology Sensory Neural and Behavioral Physiology	Royal Zoological Society of New South Wales Forum Too Close for Comfort	Revista de Biological Tropical	Transnational Environmental Law
Japanese Journal of Zoo and Wildlife Medicine			Progress in Oceanography	Journal of Comparative Physiology B-Biochemical Systemic and Environmental Physiology	Russian Journal of Theriology	Russian Journal of Ecology	World Trade Review
Journal of Anatomy			Regional Studies in Marine Science	Journal of the History of Biology	Semina Ciencias Agrarias	South African Journal of Wildlife Research	Journal of Anatomy
Journal of Chromatography B-Analytical Technologies in the Biomedical and Life Sciences		Report of the International Whaling Commission	Journal of Theoretical Biology	UK, Agricultural and Food Research Council	Wildlife Research		Journal of Chromatography B-Analytical Technologies in the Biomedical and Life Sciences
Journal of Comparative Pathology			Revista de Biologia Marina y Oceanografia	Mikrobiologiya	Wiadomosci Zootechniczne	Wildlife Society Bulletin	
Journal of Comparative Psychology			Scientific Reports of the Whales Research Institute Tokyo	Molecular Biology and Evolution	Zoo Biology		
Journal of Endocrinology			Sea Lions of the World - 22nd Lowell Wakefield Fisheries Symposium on Sea Lions of the World	Molecular Ecology	Zoologia		
Journal of Helminthology			South African Journal of Marine Science	Nature Genetics	Zoological Science		
Journal of Investigative Dermatology			Transactions of the American Fisheries Society	Nutrition and Metabolic Insights	Zoologichesky Zhurnal		
Journal of Neurophysiology			Vestnik of Astrakhan State Technical University. Series: Fishing Industry	PeerJ			
Journal of the American Veterinary Medical Association				Perceptual and Motor Skills			
Journal of Veterinary Behaviour-Clinical Applications and Research			Physiological Genomics				
Journal of Veterinary Diagnostic Investigation				Physiological Reports			
Journal of Veterinary Medical Science				Physiology and Behaviour			
Journal of Veterinary Medicine Japan				Polar Biology			
Journal of Wildlife Diseases				Proceedings of the Royal			

	Society B-Biological Sciences
Journal of Zoo and Wildlife Medicine	Zhurnal Evolyutsionnoi Biokhimii i Fiziologii
Large Animals Review	
Lucrari Stiintifice - Universitatea de Stiinte Agricole a Banatului Timisoara, Medicina Veterinara	
Medical Hypotheses	
Medicine of Australian Mammals	
Neuroimmunomodulation	
Neuroscience	
Parasites and Vectors	
Parasitology	
Parasitology Research	
Proceedings of the International Conference on Diseases of Zoo and Wild Animals, May 13th-16th, 2015, Barcelona, Spain	
Proceedings of the International Conference on Diseases of Zoo and Wild Animals, Warsaw, Poland, 28-31 May 2014	
Proceedings of the International Scientific Meeting of anatomy and physiology, fundamentals of medicine, Zagreb, Croatia, 12-13 June, 2009	
Proceedings of the NAVC Conference, 17-21 January 2015, Orlando, Florida, USA. Volume 29, Small animal and exotics edition, Book 1 & Book 2	
Redvet	
Revista Cientifica-Facultad de Ciencias Veterinarias	
Revista Portuguesa de Ciencias Veterinarias	
Sbornik Nauchnykh Trudov Moskovskaya Veterinarnaya Akademiya	
Taiwan Veterinary Journal	
Tijdschrift voor Diergeneeskunde	
Veterinaria Mexico	
Veterinary Clinical	

Pathology
Veterinary Immunology and Immunopathology
Veterinary Journal
Veterinary Parasitology
Veterinary Record
Vlaams Diergeneeskundig Tijdschrift
Zhurnal Vysshei Nervnoi Deyatelnosti Imeni I.P. Pavlova
Zoo and Wild Animal Medicine
Zycie Weterynaryjne
